# Comparison of the impact of autologous cell therapy and conservative standard treatment on tissue oxygen supply and course of the diabetic foot in patients with chronic limb-threatening ischemia: A randomized controlled trial

**DOI:** 10.3389/fendo.2022.888809

**Published:** 2022-08-29

**Authors:** Michal Dubský, Jitka Husáková, Robert Bem, Alexandra Jirkovská, Andrea Němcová, Vladimíra Fejfarová, Karol Sutoris, Michal Kahle, Edward B. Jude

**Affiliations:** ^1^ Diabetes Centre, Institute for Clinical and Experimental Medicine, Prague, Czechia; ^2^ First Faculty of Medicine, Charles University, Prague, Czechia; ^3^ Clinic of Transplant Surgery, Institute for Clinical and Experimental Medicine, Prague, Czechia; ^4^ Department of Data Analysis, Statistics and Artificial Intelligence, Institute for Clinical and Experimental Medicine, Prague, Czechia; ^5^ Tameside and Glossop Integrated Care NHS Foundation Trust and University of Manchester, Ashton under Lyne, United Kingdom

**Keywords:** chronic limb-threatening ischemia, autologous cell therapy, diabetic foot, revascularization, major amputation of lower extremity

## Abstract

**Background:**

Autologous cell therapy (ACT) is a new treatment method for patients with diabetes and no-option chronic limb-threatening ischemia (NO-CLTI). We aimed to assess the impact of ACT on NO-CLTI in comparison with standard treatment (ST) in a randomized controlled trial.

**Methods:**

Diabetic patients with NO-CLTI were randomized to receive either ACT (n=21) or ST (n=19). After 12 weeks, those in the ST group, who did not improve were treated with ACT. The effect of ACT on ischemia and wound healing was assessed by changes in transcutaneous oxygen pressure (TcPO_2_) and the number of healed patients at 12 weeks. Pain was evaluated by Visual Analogue Scale (VAS). Amputation rates and amputation-free survival (AFS) were assessed in both groups.

**Results:**

During the first 12 weeks, TcPO_2_ increased in the ACT group from 20.8 ± 9.6 to 41.9 ± 18.3 mm Hg (p=0.005) whereas there was no change in the ST group (from 21.2 ± 11.4 to 23.9 ± 13.5 mm Hg). Difference in TcPO_2_ in the ACT group compared to ST group was 21.1 mm Hg (p=0.034) after 12 weeks. In the period from week 12 to week 24, when ST group received ACT, the TcPO_2_ in this group increased from 20.1 ± 13.9 to 41.9 ± 14.8 (p=0.005) while it did not change significantly in the ACT in this period. At 24 weeks, there was no significant difference in mean TcPO_2_ between the two groups. Wound healing was greater at 12 weeks in the ACT group compared to the ST group (5/16 vs. 0/13, p=0.048). Pain measured using VAS was reduced in the ACT group after 12 weeks compared to the baseline, and the difference in scores was again significant (p<0.001), but not in the ST group. There was no difference in rates of major amputation and AFS between ACT and ST groups at 12 weeks.

**Conclusions:**

This study has showed that ACT treatment in patients with no-option CLTI and diabetic foot significantly improved limb ischemia and wound healing after 12 weeks compared to conservative standard therapy. Larger randomized controlled trials are needed to study the benefits of ACT in patients with NO-CLTI and diabetic foot disease.

**Trial registration:**

The trial was registered in the National Board of Health (EudraCT 2016-001397-15).

## Introduction

Chronic limb-threatening ischemia (CLTI) is a new term used for the end stage of peripheral arterial disease instead of the older term “critical limb ischemia”. Management of CLTI has been recently summarized in a guideline document, ‘Global Vascular Guidelines’ (1). This paper concludes that there is strong evidence to support that all patients with CLTI should use antiplatelet agents, statins, especially after any revascularization; and moderate evidence has been reported for the use of clopidogrel, rivaroxaban or vitamin K antagonists´. These guidelines also suggest that the choice of the optimal revascularization technique of CLTI should be decided based on PLAN concept (Patient risk estimation, Limb staging, ANatomic pattern of the disease) (1) and by local expertise (2). Obstruction of the common femoral artery or long occlusions above the knee are usually suitable for surgical approach (3). There are still many patients with the most severe stages of CLTI who are not suitable for any kind of standard revascularization, and suffer from high rates of major amputation and mortality. These patients are usually classed as “no-option CLTI” (4).

Common revascularization technique of CLTI includes percutaneous transluminal angiography (PTA) or bypass with increasing preference for endovascular treatment, whereas these methods are not sufficient in all patients and some patients can develop stenosis or occlusion after these procedures (1, 5). Autologous cell therapy (ACT) is a new experimental treatment and is being increasingly researched as an option in those with CLTI (6). It has been suggested that if it is applied appropriately and followed by other comprehensive therapy (7–9) these “no-option” patients could have a therapeutic benefit from ACT despite the fact that the level of evidence for the effect of this treatment in vascular guidelines is described as low to moderate (1, 10).

Recently published meta-analyses and reviews concluded that ACT is a safe method for the treatment of CLTI in patients with diabetic foot disease and significantly increases main ischemia parameters including ankle-brachial index (ABI), toe-brachial index (TBI), transcutaneous oxygen pressure (TcPO_2_) and laser doppler flowmetry (7, 9, 11, 12). The results from randomized controlled trials on limb salvage and the effect of ACT on amputation-free survival (AFS) are variable. Some of the studies reported a decrease in amputation rates after cell therapy (13–15), whereas others reported no significant impact of ACT on major amputation or AFS (16, 17).

The aim of our study was to compare the effect on tissue oxygenation and clinical outcome of ACT versus conservative standard treatment (ST) in patients with diabetic foot not eligible for standard revascularization (NO-CLTI). Other objectives of the study were quality of life assessment and side effects of ACT.

## Research design and methods

Patients with no-option CLTI (defined as the presence of non-healing ulcers or gangrene with objectively proven arterial occlusive disease and TcPO_2_ < 35 mm Hg and not eligible for standard revascularization - PTA or bypass) and diabetic foot disease treated in our foot center between 2016 and 2019 were included in this study according to inclusion and exclusion criteria described in **Table 1**. All patients fulfilling the inclusion criteria were discussed at the multidisciplinary team meeting where foot care specialists (diabetologist, interventional radiologist, vascular surgeon and general surgeon) decided if the patient with CTLI was not a candidate for revascularization (“no-option” CLTI) and could be included in the study. After informed consent the patient was prescreened for the clinical trial in our foot clinic and underwent the following examinations: TcPO_2_, peripheral angiogram, blood tests (full blood count, C-reactive protein, creatinine), standard oncological screening (tumor markers, blood in stool and gynecology assessment and mammogram in women), evaluation of the foot status and fundus examination of the eyes. When pre-screening examinations revealed no contraindication, all other scheduled examinations before the inclusion in the study were done: electrocardiogram, X-ray of chest and foot, abdominal ultrasound, tests for coagulation disorders, renal and liver function tests, optional duplex ultrasound of lower limb (focusing on infrapopliteal arteries) and hematological examination.

**Table 1:** Inclusion and exclusion criteria.

Inclusion criteria	Exclusion criteria
1. Diabetic foot (ulcer distal from ankle) and/or status after minor amputation, in accordance with international classification TEXAS 2-3 (C-D), Wagner 2-4, 2. Presence of CLTI – ulcers or gangrene attributable to objectively proven arterial occlusive disease (TcPO_2_ below 35 mm Hg), non-eligibility for standard revascularization (PTA or by-pass), 3. Age 18-90 years, 4. Diabetes mellitus type 1 or 2.	1. Severe active deep infection of the foot, 2. Deep vein thrombosis in previous 6 months in lower limbs, 3. Severe limb edema that rule out intramuscular injection of cell suspension, 4. Severe non-treated diabetic retinopathy requiring acutely a laser therapy, 5. Severe hematological disease, 6. Diagnosed neoplastic process of any organ, 7. Life expectancy less than 6 months, 8. Contraindication of general anesthesia, 9. Females of childbearing potential must be willing to use a highly effective method of contraception.

**Figure 1:**
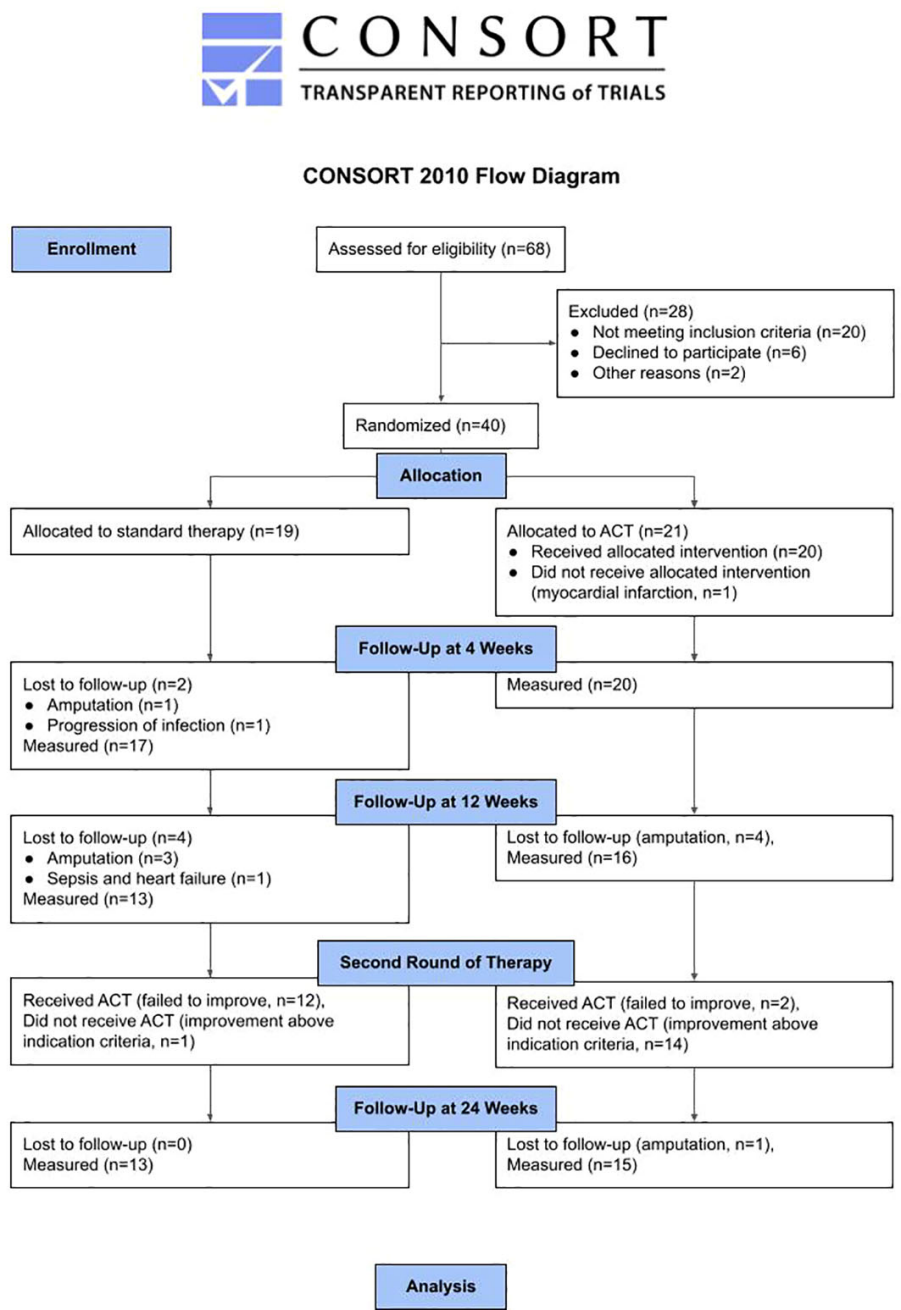
CONSORT flow diagram of the study.

After the selection visit (**Figure 1**), there was a two-week follow-on time and if the TcPO_2_ at the end of this period was below 35 mm Hg the patient was randomized (1:1) to either ACT treatment plus standard care or standard care only. Standard therapy of no-option CLTI patients consists of medication (usually antiplatelet or anticoagulant therapy, analgesic therapy), management of infection, off-loading and local treatment of diabetic foot. Forty patients were randomly assigned by means of computerized block randomization with variable block sizes to two treatment groups; 21 patients were assigned to ACT and 19 to ST. Participants were enrolled and assigned to interventions by the principal investigator. One patient dropped out of the ACT group due to myocardial infarction shortly after randomization. In the ST group, one patient dropped out at 4 weeks due to progression of infection and another one dropped out at 12 weeks follow-up due to sepsis and heart failure. Because of major amputation, 5 patients dropped out of the ACT group and 4 patients dropped out of the ST group.

The effect of ACT was compared with ST after 12 weeks and patients originally receiving standard treatment were treated with ACT at 12 weeks. One patient in the ST group improved at 12 weeks with TcPO_2_ > 35 mm Hg and did not receive ACT. Two of the ACT group patients who failed to improve at 12 weeks to TcPO_2_ > 35 mm Hg received a second treatment with ACT.

Cell suspension was prepared in the Advanced Therapy Medicinal Product technique in accordance with European Medicines Agency guidelines and the State Institute for Drug Control. Bone marrow-derived mononuclear cells (BMMNC) was prepared from 250 ml of bone marrow harvested from the iliac crest in the operation theatre using Jamshidi needle and mixed with diluted heparin and acid citrate dextrose solution as anticoagulant agents. Then the bone marrow was filtered, injected into four 60 mL centrifugation containers and centrifuged at 4000 rpm for 15 minutes by Harvest Smart PReP2 (Harvest Technologies Corporation, Plymouth, MA, USA). Forty mL of final cell therapy product (CTP) was injected under sterile conditions in series of 1 ml punctures into the muscles of the affected lower limb and to the edges of the wound or gangrene. Patients received the cell therapy product the same day as bone marrow harvesting was performed.

The CTP was analyzed by means of CD surface markers using flow cytometry to assess the quality of the cell suspension. Besides widely used anti-CD34 we measured allophycocyanin labelled anti-CD45 and anti-CD146, phycoerythrin labelled anti-CD105 and anti-CD73 and fluorescein isothiocyanate labelled anti-CD90 and anti-CD45.

Participants were followed up in the foot clinic with measurements at 2, 4 and 12 weeks. Following variables were assessed: TcPO_2_, subjective pain sensation, quality of life (using standardized questionnaires), wound healing, amputation rates, AFS and adverse events. TcPO_2_ was measured using a TCM400 monitor (RadioMeter, Brea, California, USA) and performed on the dorsum of the foot between the 1st and 2nd metatarsal heads with room temperature standardized (22°C) and the patient lying supine for 40 minutes.

The physician who evaluated the TcPO_2_ changes was blinded and not included in the study (and was from a different hospital to the main investigator). Team decision-making on the need for amputation was made by clinicians not involved in the study who were also blinded to treatment received.

The study was approved by the local ethics committee and was carried out in agreement with the protocol, ICG Guideline E6 on Good Clinical Practice, Declaration of Helsinki and applicable law of the Czech Republic.

All participants were treated in our foot center during the follow-up in accordance with national standards. Those requiring pressure offloading received therapeutic shoes, orthotics or a total contact cast. For infected ulcers we prescribed appropriate antibiotic therapy taking into account their renal function.

Wound healing (defined as complete re-epithelization of the wound for at least 4 weeks) and the change of total wound size was assessed from photographs – pictures were taken by Samsung Galaxy A7 (standardized distance from the wound was 20 cm) and directly uploaded to our medical record system. The wound area was outlined manually, scale was set by the photographed ruler and area was calculated using Image J (https://fiji.sc).

Quality of life was evaluated by two standardized questionnaires, EuroQoL-5 dimension (EQ-5D) and Short-form (36) Health Survey (SF-36), and by a special questionnaire focused on pain using Visual Analogue Scale (VAS, scale 1-10) (18). The inflow/outflow status of the vasculature at baseline was assessed by the Graziani classification of infrapopliteal arteries (**Table 2**).

**Table 2:** Baseline characteristics of the patients.

Parameter	ACT (n=21)	ST (n=19)	p value
***Patient-related factors***			
Age [years]	69.9 ± 9.7	66.2 ± 10.1	0.25
Female sex	2 (9.5)	2 (10.5)	1.0
Diabetes duration [years]	22.0 ± 7.8	22.6 ± 9.2	0.71
HbA1c [mmol/mol]	59.9 ± 13.3	58.4 ± 16.7	0.68
Coronary heart disease	15 (71.4)	14 (73.7)	1.0
Hemodialysis	3 (14.3)	5 (26.3)	0.44
Immunosuppressive therapy	4 (19.0)	4 (21.1)	1.0
Diabetic neuropathy	20 (95.2)	18 (94.7)	1.0
Hypertension	20 (95.2)	18 (94.7)	1.0
***Limb-related factors***			
TcPO_2_ [mmHg]	18.7 ± 9.9	21.1 ± 11.4	0.42
Ulcer/gangrene duration [months]	8.0 ± 4.2	9.4 ± 4.7	0.32
Resistant microbes	4 (19.0)	4 (21.1)	1.0
Osteomyelitis	6 (28.6)	6 (31.6)	1.0
CRP [mg/L]	8.9 ± 10.1	20.4 ± 21.1	0.058
Rutherford category			
4	2 (9.5)	1 (5.3)	1.0
5	19 (91.5)	17 (89.4)	1.0
6	0 (0.0)	1 (5.3)	0.48
Graziani stage			
4	4 (19.0)	1 (5.3)	0.35
5	10 (47.6)	12 (63.2)	0.36
6	6 (28.6)	6 (31.6)	1.0
7	1 (4.8)	0 (0.0)	1.0
WIfI – Clinical stage			0.35
3	6 (28.6)	3 (15.8)	
4	15 (71.4)	16 (84.2)	
WIfI – Wound			0.81
1	4 (19.0)	3 (15.8)	
2	15 (71.4)	14 (73.7)	
3	2 (9.5)	2 (10.5)	
WIfI – Ischemia			1.0
3	21 (100.0)	19 (100.0)	
WIfI – Infection			0.24
0	11 (52.4)	7 (36.8)	
1	9 (42.9)	9 (47.4)	
2	1 (4.8)	3 (15.8)	

### Statistical methods

We have used the Python ecosystem to perform statistical analysis. A list of specific packages and versions used together with source data and a reproducible notebook are available as supplement to this article. All observed variables are expressed as mean ± standard deviation or percentages. The significance level of all tests was 5%. Estimation of the sample size of the trial was based on the best available evidence at trial initiation, with expected amputation rates between 20-30% of patients with CLTI. With a proposed number of 20 cases at baseline in each group expected to drop down to 15 because of amputations, significance level of 5%, SD of TcPO_2_ 11 mm Hg (19) and minimum meaningful difference between groups of 15 mm Hg the power of unpaired t-test to reject the null hypothesis would be 95%. The statistical analyses of quantitative variables were performed using Welch two-sample t-tests, paired t-tests, Mann-Whitney U test and Wilcoxon signed rank test. Binary variables (amputation, AFS, number of healed patients, SF-36) were analyzed *via* Fisher’s exact test and McNemar test. To test the statistical significance of the differences in TcPO_2_ measurements we have used nonparametric tests with Bonferroni-Holm correction for multiple testing (6 tests).

## Results

There was no difference between study groups in baseline characteristics including age, gender, control and duration of diabetes, treatment, and presence of comorbidities, as well as foot and ischemia parameters (**Table 2**).

**Figure 2:**
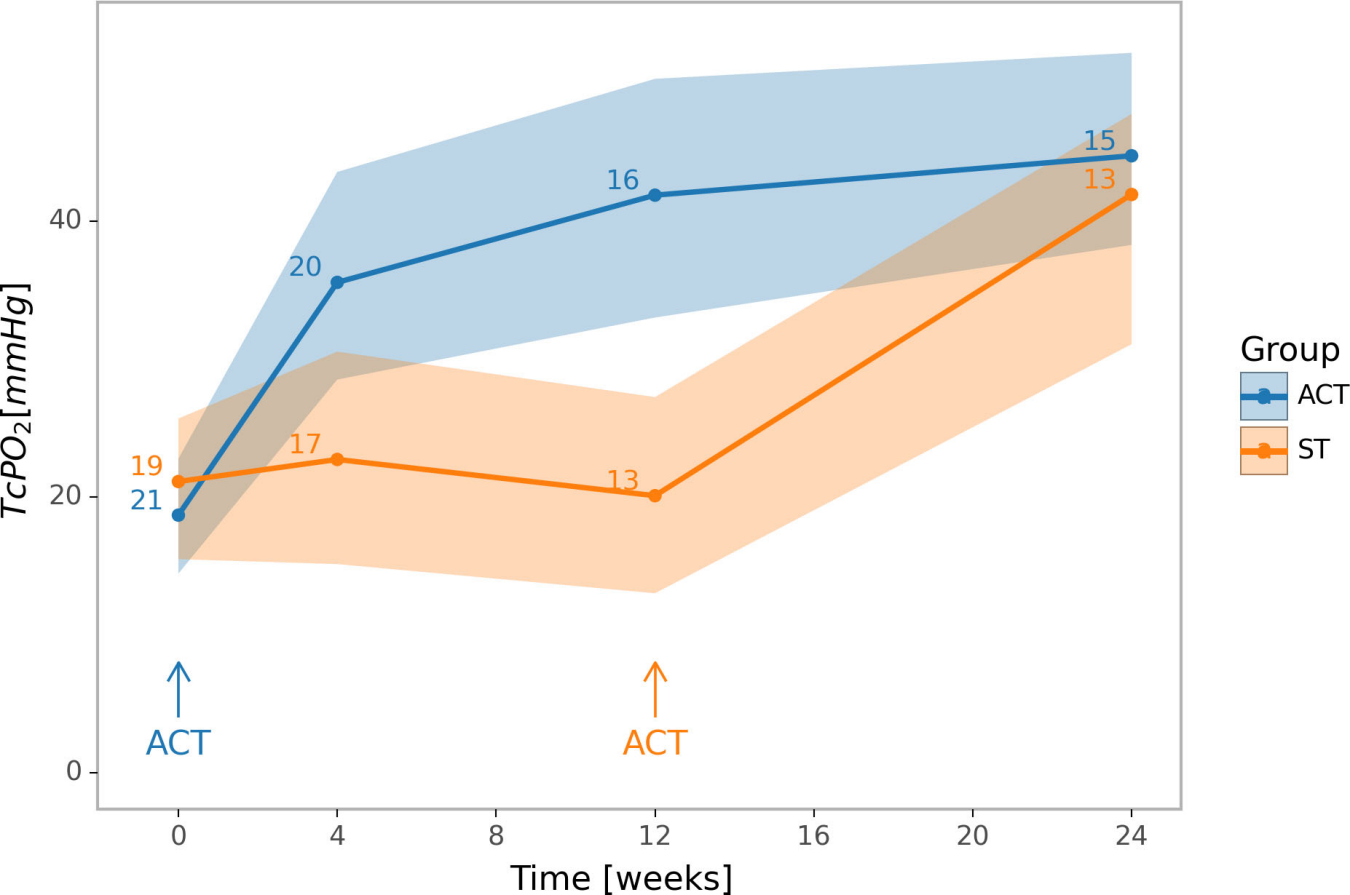
Changes in limb oxygenation. Means and 95% confidence intervals of TcPO_2_. Blue colour represents the group initially treated by ACT, orange colour represents the group initially on standard therapy. In the week 12 of the experiment, a difference between ACT and ST groups in TcPO_2_ was significant; within the ACT group, the TcPO_2_ differs significantly in the week 12 from its baseline level; within the ST group, the TcPO_2_ differs significantly in the week 24 from its level in the 12th week. Error bars indicate 95% confidence intervals.

The mean and standard deviation for TcPO_2_ in both groups at each measured time are shown in **Table 3**. The TcPO_2_ (means and their 95% confidence intervals) are illustrated in **Figure 2**.

**Table 3:** Descriptive statistics of TcPO_2_ measurements.

	Standard therapy first		Autologous cell therapy first	
week	n	mean ± sd	n	mean ± sd
0	19	21.1 ± 11.4	21	18.7 ± 9.9
4	17	22.7 ± 17.2	20	35.5 ± 17.5
12*	13	20.1 ± 13.9	16	41.9 ± 18.3
24	13	41.9 ± 14.8	15	44.7 ± 14.2

*Patients in ST group received ACT at 12 weeks

The TcPO_2_ increased from 20.8 ± 9.6 to 41.9 ± 18.3 mm Hg (p = 0.005) during the first 12 weeks in the ACT group but did not change significantly in the ST group over the same time period. After 12 weeks the difference between groups was 21.8 mm Hg (p = 0.034). At this time point patients in the ST group received ACT and TcPO_2_ measured at week 24 in both groups. The TcPO_2_ in the ST group post-ACT increased from 20.1 ± 13.9 to 41.9 ± 14.8 (p = 0.005) but did not change significantly in the ACT group from 12 to 24 weeks. At 24 weeks, there was no significant difference in mean TcPO_2_ between the two groups.

We observed a significant difference in the number of healed ulcers between ACT and ST groups in patients who survived with preserved limb at 12 weeks (5/16 [31.3%] vs. 0/13 [0%], p= 0.048, Fisher exact test). Wound area reduced significantly in the ACT group (from 36.3 ± 14.4 to 24.2 ± 8.3 mm^2^) compared to the ST group (31.9 ± 15.6 to 31.1 ± 12.7 mm^2^) at 12 weeks (area reduction rate was 12.1 ± 5.2 vs. 0.8 ± 1.7 mm^2^, p < 0.001). There was no difference in minor and major amputations or AFS between the two groups at 12 weeks.

**Figure 3:**
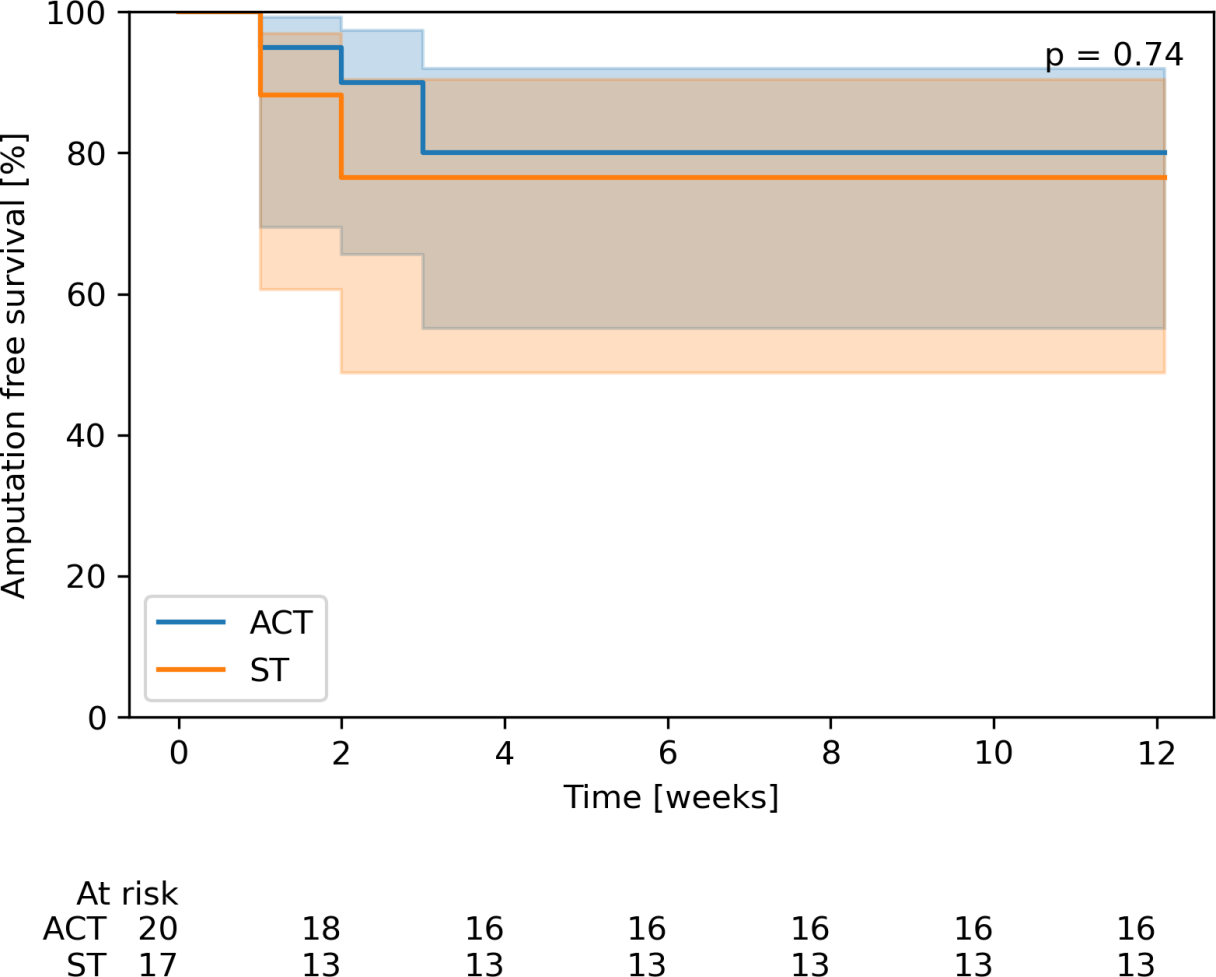
Comparison of amputation-free survival with 95% confidence intervals. Blue colour represents the group initially treated by ACT, orange colour represents the group initially on standard therapy.

Major amputation rate (4/20 vs. 4/17) and AFS (16/20 vs. 13/17) did not differ significantly after 12 weeks between ACT and ST groups. AFS was counted using Kaplan-Meier method with no significant difference (p = 0.74, **Figure 3**). Main indication for major amputation in the ACT group was progression of infection (3/4 [75%]); and in the ST group it was the progression of ischemia (3/4 [75%]).

Patients initially treated by ACT experienced a significant decrease in pain measured by VAS during the first 12 weeks (from 5.5±2.5 to 2.3±2.1, p = 0.002). However, patients in the ST group experienced a significant increase in pain (from 5.0±2.2 to 5.9±1.7, p = 0.003).

There was a significant increase in quality of life 12 weeks after ACT on EQ-5D in comparison to baseline EQ-5D (from 48.0 ± 12.0 to 69.6 ± 13.2%, p < 0.001). The change of the quality of life in the ST group was not significant (from 47.1 ± 10.2 to 41.8 ± 9.6%). Using SF-36 the ACT group described their health status as “very bad” in 90.5% at baseline and only 15% after 12 weeks (p< 0.001 for McNemar test). In the ST group it was 100% patients at both baseline and after 12 weeks.

To assess the quality of the CTP we analyzed cell surface markers using flow cytometry. The cells that were characterized by flow cytometry as CD45^-^CD90^+^CD73^+^ and CD105^+^ were described as mesenchymal stem cells (MSCs), CD45^+^ and CD146^-^ were defined as myeloid angiogenic cells (MACs) and CD45^-^ and CD146^+^ cells were analyzed as endothelial colony forming cells (ECFCs). MACs represented approximately 23% of CD45^+^ cells, ECFCs 1.3% of CD45^-^ and MSCs more than 90% of CD45^-^ BM-SCs. This finding suggested the potential role of MACs as an important cell lineage in limb revascularization.

Adverse events after ACT were noted in a small number of participants: bleeding after trepanation of bone marrow in 1 patient (5%) and temporary worsening of limb edema after injection of cell suspension in 1 participant (5%).

## Discussion

In this randomized trial we have shown that autologous cell therapy can lead to an improvement in limb ischemia as measured by TcPO_2_ in people with diabetes and no-option CLTI and also improved healing of foot ulcers during the short period of 12 weeks from treatment.

In our previous studies we have shown that cell therapy of CLTI was safe, resulted in an increase in TcPO_2_ and was comparable with repeated percutaneous transluminal angioplasty (19, 20). The benefit of ACT on limb ischemia and salvage has been reported in several randomized controlled trials (14, 21, 22) and meta-analyses in those with and without diabetes (23, 24). On the other hand, some studies reported no significant benefit of ACT on amputation rates (16, 17). This discrepancy can be caused by different factors that could affect major amputation besides ischemia (progression of infection, severe osteomyelitis, patient’s choice) and therefore we focused more on the change in ischemia parameters (mainly TcPO_2_ since ABI is less conclusive and more than 75% of patients in our study had severe medial sclerosis which can lead to falsely elevated ABI). Other possible problems in many studies of CLTI cell therapy is that they are very heterogeneous; they differ in cell type (BMMNC, peripheral blood progenitor cells, mesenchymal stem cells, amniotic cells), cell source (bone marrow, peripheral blood, adipose tissue, umbilical cord), host source (autologous, allogeneic), health of the host source (healthy or diseased), cell manipulation (culture, preconditioning), route of delivery (intraarterial, intramuscular, intravenous) and dosing frequency (single or multiple) (8, 11, 25, 26).

One of the consistent endpoints for the assessment of improvement in circulation is by TcPO_2_ – it is considered a standard method of non-invasive assessment of CLTI (27), because it evaluates microcirculation in the skin of the foot and therefore is less affected by the presence of neuropathy and other diabetes complications and an indicator of tissue perfusion. This variable increased significantly after ACT, but no change in the standard therapy group was seen. These findings are in conformity with published data, where most studies have reported an increase in TcPO_2_ after cell therapy in patients with CLTI (28–30).

Amputation rates (major and minor) and AFS were not significantly different after 12 weeks between ACT and standard therapy groups and maybe related to short follow-up (12 weeks). These findings are in accordance with reported very high mortality and amputation rates among the patients with no-option CLTI (1, 4). In our previous studies we also observed a high mortality in no-option CLTI patients that can strongly affect the AFS (23).

Even though the amputation rates were not different, we observed a significant impact of ACT on patients’ quality of life measured by different standardized questionnaires. Most of the patients described decreased pain, reduced depression and improvement in well-being and general health condition.

We believe that cells responsible for revascularization could potentially be MACs because they represented more than 20% of all CD45+ cells. This cell lineage is similar to early endothelial progenitor cells and has been shown to form vascular endothelium (31). Cytokines and growth factors can also play a key role in the revascularization effect of ACT. Therefore we analyzed serum levels of angiogenic cytokines in our previous studies to prove that ACT caused no systemic vasculogenesis. We did not observe any increase in serum levels of proangiogeneic cytokines after ACT in our previous studies (32), on the other hand we demonstrated a significant increase in endogenous inhibitor of angiogenesis endostatin at 1 and 3 months after ACT, these results might explain the possible negative feedback reaction on the local vasculogenesis in the limb (33).

We did not observe any serious adverse events after cell therapy that would lead to any deterioration in quality of life. Cell treatment of CLTI by the use of bone marrow-derived precursor cells seems to be a safe method without any major concerns about dedifferentiation or tumorigenesis (29).

The limitation of our study was the relatively small number of participants in each group. We did not use placebo as a comparator because with current level of evidence of cell therapy we considered it unethical to use intramuscular saline injections in patients with no-option CLTI. Advantages of our study were well defined inclusion criteria, and standard methods used for assessment with regular visits to the foot clinic with prompt opportunity for hospitalization and team decision-making on the need for amputation by experts not involved in the study. All people were treated in a single center by standard methods, and all groups were comparable in main demographic characteristics.

## Conclusion

Our randomized controlled trial showed that autologous cell therapy in patients with no-option CLTI and diabetic foot significantly improved limb ischemia and foot ulcer healing and decreased pain when compared to standard conservative therapy, but did not influence amputation rates or amputation-free survival in short-term follow-up of 12 weeks.

## Data availability statement

The original contributions presented in the study are included in the article/**Supplementary Material**. Further inquiries can be directed to the corresponding author.

## Ethics statement

The studies involving human participants were reviewed and approved by ethics committee of IKEM and Thomayers Hospital. The patients/participants provided their written informed consent to participate in this study.

## Author contributions

MD - wrote the manuscript, patient selection, performed the procedure, analyzed data, revised the manuscript. JH - patient selection, performed the procedure. RB - performed the procedure, revised the manuscript. AJ - revised the manuscript, contributed to discussion. AN - patient selection, performed the procedure. VF - patient selection, revised the manuscript. KS - performed the procedure. MK - analyzed data, revised the manuscript. EJ - revised the manuscript, contributed to discussion. All authors contributed to the article and approved the submitted version.

## Funding

This study was supported by the Ministry of Health of the Czech Republic, grant no. 00023001 and by the project National Institute for Research of Metabolic and Cardiovascular Diseases (Programme EXCELES, Project No. LX22NPO5104) - Funded by the European Union - Next Generation EU.

## Conflict of interest

The authors declare that the research was conducted in the absence of any commercial or financial relationships that could be construed as a potential conflict of interest.

## Publisher’s note

All claims expressed in this article are solely those of the authors and do not necessarily represent those of their affiliated organizations, or those of the publisher, the editors and the reviewers. Any product that may be evaluated in this article, or claim that may be made by its manufacturer, is not guaranteed or endorsed by the publisher.
