# Supplementary material for: Comparison of the impact of autologous cell therapy and conservative standard treatment on tissue oxygen supply and course of the diabetic foot in patients with chronic limb-threatening ischemia: A randomized controlled trial
Source: Front Endocrinol (Lausanne). 2022 Aug 29;13:888809. doi: 10.3389/fendo.2022.888809 (PMC9464922; doi:10.3389/fendo.2022.888809)
Supplement: Supplementary file 1 [file DataSheet_1.pdf]

mids_SC

August 17, 2022

[1]:
```
import pandas as pd
import numpy as np
import matplotlib
from matplotlib import pyplot as plt
import plotnine as p9
# p9.options.figure_size = (6,4)
from plotnine import *
p9.theme_set(p9.theme_light)
from scipy import stats
import statsmodels as sm
import lifelines
```

[2]:
```
# install from https://github.com/michalkahle/statistikem.git
import statistikem as si
```

[3]:
```
alpha = 0.05
```

0.1 Power calculation

[4]:
```
ttp_2_samples = sm.stats.power.TTestIndPower()
es = 15 # minimal interesting effect-size
sd = 11 # from Dubsky 2013
d = es/sd
nobs = round(20 * .75)
power = ttp_2_samples.solve_power(effect_size=d, nobs1=nobs, alpha=0.05,␣
 ↪power=None)
print(f'd = {d:.3f}\nnobs = {nobs}\npower = {power:.2f}')
```

```
d = 1.364
nobs = 15
power = 0.95
```

0.2 Data loading

[5]:
```
df = pd.read_csv('source_data_3.csv')
df['Group'] = pd.Categorical(df['Group'])
df
```

[5]:

	Patient	Group	Age	Female	Cr	MDRD	CKD	HbA1c	Years_DM	CHD	…	\
0	2	ACT	82	0	95	1.15	2	61	21	1	…	
1	3	ACT	72	0	66	1.70	1	56	47	0	…	
2	4	ACT	70	0	492	0.10	5	56	13	1	…	
3	6	ACT	53	0	251	0.39	4	75	23	0	…	
4	8	ACT	72	1	82	0.99	3	47	17	0	…	
5	10	ACT	73	0	73	1.52	1	58	31	0	…	
6	13	ACT	63	0	588	0.14	5	39	23	1	…	
7	14	ACT	80	0	94	1.01	2	58	27	1	…	
8	17	ACT	64	0	81	1.39	2	64	20	1	…	
9	16	ACT	63	0	638	0.13	5	101	28	1	…	
10	18	ACT	64	0	69	1.67	1	64	12	1	…	
11	20	ACT	68	0	107	1.00	2	65	26	1	…	
12	21	ACT	85	0	73	1.46	1	61	25	1	…	
13	23	ACT	69	0	160	0.62	3	53	20	1	…	
14	25	ACT	80	0	90	1.17	2	57	20	1	…	
15	29	ACT	75	0	99	1.06	2	45	18	1	…	
16	30	ACT	51	0	95	1.21	2	46	23	1	…	
17	33	ACT	70	1	101	0.79	3	64	24	0	…	
18	34	ACT	63	0	107	1.01	2	73	11	1	…	
19	35	ACT	88	0	201	0.46	4	68	20	1	…	
20	36	ACT	62	0	135	9.90	3	46	14	0	…	
21	1	ST	64	0	65	1.77	1	45	21	1	…	
22	5	ST	70	1	792	0.07	5	58	24	0	…	
23	7	ST	63	0	179	0.56	3	56	11	1	…	
24	9	ST	67	0	83	1.18	1	46	16	1	…	
25	11	ST	84	0	119	0.85	3	47	17	1	…	
26	12	ST	68	0	94	1.08	2	62	21	1	…	
27	15	ST	68	0	94	1.16	2	109	28	1	…	
28	19	ST	65	0	721	0.11	5	69	24	0	…	
29	22	ST	71	0	519	0.13	5	65	32	1	…	
30	24	ST	56	0	542	0.16	5	54	29	1	…	
31	26	ST	66	0	225	0.42	4	61	33	1	…	
32	27	ST	42	0	138	0.86	3	44	12	1	…	
33	28	ST	60	1	66	1.60	1	79	41	0	…	
34	31	ST	67	0	388	0.23	5	64	18	1	…	
35	32	ST	70	0	80	1.39	2	64	16	0	…	
36	37	ST	68	0	151	0.67	3	39	4	1	…	
37	38	ST	50	0	189	0.54	3	35	19	0	…	
38	39	ST	72	0	168	0.59	3	47	32	1	…	
39	40	ST	87	0	125	0.79	3	66	31	1	…	

	Months_amp	Amp_12	Amp_ischemia	Amp_infection	Minor_amp	AFS_12	O2_0	\
0	NaN	0.0	0.0	0.0	0.0	1.0	3	
1	NaN	0.0	0.0	0.0	0.0	1.0	18	
2	NaN	NaN	NaN	NaN	NaN	NaN	17	
3	NaN	0.0	0.0	0.0	1.0	1.0	13	

4	NaN	0.0	0.0	0.0	0.0	1.0	18
5	NaN	0.0	0.0	0.0	1.0	1.0	14
6	NaN	0.0	0.0	0.0	0.0	1.0	19
7	3.0	1.0	0.0	1.0	0.0	0.0	7
8	7.0	0.0	0.0	0.0	1.0	1.0	33
9	NaN	0.0	0.0	0.0	0.0	1.0	23
10	2.0	1.0	0.0	1.0	0.0	0.0	12
11	3.0	1.0	0.0	1.0	0.0	0.0	22
12	NaN	0.0	0.0	0.0	0.0	1.0	28
13	1.0	1.0	1.0	0.0	0.0	0.0	1
14	NaN	0.0	0.0	0.0	1.0	1.0	28
15	NaN	0.0	0.0	0.0	0.0	1.0	31
16	NaN	0.0	0.0	0.0	0.0	1.0	32
17	5.0	0.0	0.0	0.0	1.0	1.0	20
18	NaN	0.0	0.0	0.0	0.0	1.0	34
19	NaN	0.0	0.0	0.0	0.0	1.0	6
20	NaN	0.0	0.0	0.0	0.0	1.0	13
21	NaN	0.0	0.0	0.0	1.0	1.0	6
22	NaN	0.0	0.0	0.0	0.0	1.0	34
23	NaN	0.0	0.0	0.0	1.0	1.0	22
24	NaN	NaN	NaN	NaN	NaN	NaN	6
25	NaN	0.0	0.0	0.0	0.0	1.0	10
26	2.0	1.0	1.0	0.0	0.0	0.0	28
27	6.0	0.0	0.0	0.0	1.0	1.0	18
28	2.0	1.0	0.0	1.0	0.0	0.0	32
29	1.0	1.0	1.0	0.0	0.0	0.0	33
30	NaN	0.0	0.0	0.0	0.0	1.0	24
31	NaN	0.0	0.0	0.0	1.0	1.0	5
32	NaN	0.0	0.0	0.0	0.0	1.0	32
33	1.0	1.0	1.0	0.0	0.0	0.0	5
34	NaN	0.0	0.0	0.0	0.0	1.0	27
35	9.0	0.0	0.0	0.0	0.0	1.0	23
36	NaN	0.0	0.0	0.0	0.0	1.0	30
37	NaN	0.0	0.0	0.0	1.0	1.0	34
38	NaN	0.0	0.0	0.0	0.0	1.0	4
39	NaN	NaN	NaN	NaN	NaN	NaN	28

	O2_4	O2_12	O2_24
0	48.0	45.0	49.0
1	73.0	2.0	26.0
2	NaN	NaN	NaN
3	60.0	57.0	41.0
4	41.0	39.0	48.0
5	16.0	30.0	43.0
6	40.0	33.0	49.0
7	26.0	NaN	NaN
8	22.0	49.0	59.0

```
9   53.0   51.0   42.0
10   3.0    NaN    NaN
11  24.0    NaN    NaN
12  32.0   49.0   56.0
13  31.0    NaN    NaN
14  48.0   30.0   30.0
15  60.0   75.0   77.0
16  30.0   68.0   24.0
17  28.0   30.0    NaN
18  21.0   25.0   27.0
19  39.0   58.0   50.0
20  16.0   29.0   50.0
21   3.0    6.0   40.0
22  38.0   13.0   52.0
23   4.0    3.0   60.0
24   7.0    NaN    NaN
25  39.0   26.0   38.0
26   3.0    NaN    NaN
27   2.0   17.0   30.0
28  26.0    NaN    NaN
29   NaN    NaN    NaN
30  30.0   35.0   54.0
31  18.0   12.0   48.0
32  34.0   35.0   50.0
33  16.0    NaN    NaN
34   3.0    5.0   37.0
35  53.0    3.0    2.0
36  46.0   34.0   53.0
37  40.0   37.0   36.0
38  24.0   35.0   45.0
39   NaN    NaN    NaN

[40 rows x 50 columns]
```

0.3 Baseline characteristics

[6]:
```
cat_columns = ['Rutherford', 'Graziani', 'WIfI_clin', 'WIfI_wound',␣
 ↪'WIfI_ischemia', 'WIfI_infection']
dummies = pd.get_dummies(df[cat_columns], columns=cat_columns)
data_table_1 = pd.concat([df, dummies], axis=1)
```

[7]:
```
table_spec = [
    ['Patient-related factors', None],
    ['Age - years', 'Age'],
    ['Female sex', 'Female'],
    ['Diabetes duration - years', 'Years_DM'],
    ['HbA1c - mmol/mol', 'HbA1c'],
```

```
    ['Coronary heart disease', 'CHD'],
    ['Hemodialysis', 'HD'],
    ['Immunosuppressive therapy', 'IS'],
    ['Diabetic neuropathy', 'DN'],
    ['Hypertension', 'HT'],

    ['Limb-related factors', None],
    ['$TcPO_2$ - mmHg', 'O2_0'],
    ['Ulcer/gangrene duration - years', 'Ulcer duration'],
    ['Resistant microbes', 'RMB'],
    ['Osteomyelitis', 'OM'],
    ['CRP - mg/L', 'CRP'],


    ['Rutherford category', 'Rutherford', 'categorical'],
    ['4', 'Rutherford_4'],
    ['5', 'Rutherford_5'],
    ['6', 'Rutherford_6'],
    ['Graziani stage', 'Graziani', 'categorical'],
    ['4', 'Graziani_4'],
    ['5', 'Graziani_5'],
    ['6', 'Graziani_6'],
    ['7', 'Graziani_7'],
    ['WIfI - Clinical stage', 'WIfI_clin', 'categorical'],
    ['3', 'WIfI_clin_3'],
    ['4', 'WIfI_clin_4'],
    ['WIfI - Wound', 'WIfI_wound', 'categorical'],
    ['1', 'WIfI_wound_1'],
    ['2', 'WIfI_wound_2'],
    ['3', 'WIfI_wound_3'],
    ['WIfI - Ischemia', 'WIfI_ischemia', 'categorical'],
    ['3', 'WIfI_ischemia_3'],
    ['WIfI - Infection', 'WIfI_infection', 'categorical'],
    ['0', 'WIfI_infection_0'],
    ['1', 'WIfI_infection_1'],
    ['2', 'WIfI_infection_2'],
]

plot = False

def table_row(label, var, scale=None):
    res = {'Parameter' : label, 'ACT':'', 'ST':'', 'p value':''}
    if var is not None:
        s = data_table_1[var]
        scale = scale or si._guess_scale(s)
        for rand in ['ACT', 'ST']:
            data = s.loc[df['Group'] == rand]
```

```
            if scale == 'continuous':
                res[rand] = f'{data.mean():.1f} ± {data.std(ddof=1):.1f}'
            elif scale == 'categorical':
                res[rand] = ''
            elif scale == 'binary':
                res[rand] = f'{data.sum()} ({data.sum()/len(data)*100:.1f})'
        tt = si.compare_one(var, 'Group', data_table_1, scale=scale, plot=plot)
        res['p value'] = format_p(tt['p'])
        res['test'] = tt['test']
    return res

def format_p(p):
    if p == 1.0:
         return '1.0'
    elif p > 0.2:
        return f'{p:.2f}'.lstrip('0')
    elif p < 0.001:
        return '<.001'
    else:
        return f'{p:.3f}'.lstrip('0')

table2 = []
for row in table_spec:
    res = table_row(*row)
    table2.append(res)

pd.DataFrame(table2).set_index('Parameter')
```

```
/home/mkah/statistikem/statistikem.py:121: UserWarning: CRP: all groups possibly
lognormal. Tests not implemented, yet!
/opt/conda/envs/py3.9/lib/python3.9/site-packages/scipy/stats/morestats.py:2603:
RuntimeWarning: invalid value encountered in double_scalars
```

[7]:

```
                                          ACT           ST p value  \
Parameter
Patient-related factors
Age - years                        69.9 ± 9.7  66.2 ± 10.1     .25
Female sex                            2 (9.5)     2 (10.5)     1.0
Diabetes duration - years          22.0 ± 7.8   22.6 ± 9.2     .71
HbA1c - mmol/mol                  59.9 ± 13.3  58.4 ± 16.7     .76
Coronary heart disease              15 (71.4)    14 (73.7)     1.0
Hemodialysis                         3 (14.3)     5 (26.3)     .44
Immunosuppressive therapy            4 (19.0)     4 (21.1)     1.0
Diabetic neuropathy                 20 (95.2)    18 (94.7)     1.0
Hypertension                        20 (95.2)    18 (94.7)     1.0
Limb-related factors
$TcPO_2$ - mmHg                    18.7 ± 9.9  21.1 ± 11.4     .42
```

Ulcer/gangrene duration - years	8.0 ± 4.2	9.4 ± 4.7	.32
Resistant microbes	4 (19.0)	4 (21.1)	1.0
Osteomyelitis	6 (28.6)	6 (31.6)	1.0
CRP - mg/L	8.9 ± 10.1	20.4 ± 21.1	.058
Rutherford category			.36
4	2 (9.5)	1 (5.3)	1.0
5	19 (90.5)	17 (89.5)	1.0
6	0 (0.0)	1 (5.3)	.48
Graziani stage			.72
4	4 (19.0)	1 (5.3)	.35
5	10 (47.6)	12 (63.2)	.36
6	6 (28.6)	6 (31.6)	1.0
7	1 (4.8)	0 (0.0)	1.0
WIfI - Clinical stage			.35
3	6 (28.6)	3 (15.8)	.46
4	15 (71.4)	16 (84.2)	.46
WIfI - Wound			.81
1	4 (19.0)	3 (15.8)	1.0
2	15 (71.4)	14 (73.7)	1.0
3	2 (9.5)	2 (10.5)	1.0
WIfI - Ischemia			1.0
3	21 (100.0)	19 (100.0)	nan
WIfI - Infection			.24
0	11 (52.4)	7 (36.8)	.36
1	9 (42.9)	9 (47.4)	1.0
2	1 (4.8)	3 (15.8)	.33

	test
Parameter	
Patient-related factors	NaN
Age - years	t
Female sex	Fisher exact
Diabetes duration - years	Mann-Whitney
HbA1c - mmol/mol	t
Coronary heart disease	Fisher exact
Hemodialysis	Fisher exact
Immunosuppressive therapy	Fisher exact
Diabetic neuropathy	Fisher exact
Hypertension	Fisher exact
Limb-related factors	NaN
$TcPO_2$ - mmHg	Mann-Whitney
Ulcer/gangrene duration - years	t
Resistant microbes	Fisher exact
Osteomyelitis	Fisher exact
CRP - mg/L	Mann-Whitney
Rutherford category	Mann-Whitney
4	Fisher exact

```
5                                  Fisher exact
6                                  Fisher exact
Graziani stage                     Mann-Whitney
4                                  Fisher exact
5                                  Fisher exact
6                                  Fisher exact
7                                  Fisher exact
WIfI - Clinical stage              Mann-Whitney
3                                  Fisher exact
4                                  Fisher exact
WIfI - Wound                       Mann-Whitney
1                                  Fisher exact
2                                  Fisher exact
3                                  Fisher exact
WIfI - Ischemia                    Mann-Whitney
3                                          None
WIfI - Infection                   Mann-Whitney
0                                  Fisher exact
1                                  Fisher exact
2                                  Fisher exact
```

[8]:

```
tests = si.compare(['CRP', 'Rutherford', 'Graziani', 'WIfI_clin', 'WIfI_wound',␣
↪'WIfI_ischemia', 'WIfI_infection'], 'Group', df)
tests
```

```
/home/mkah/statistikem/statistikem.py:121: UserWarning: CRP: all groups possibly
lognormal. Tests not implemented, yet!
```

CRP ~ Group

	ACT	ST	total
n	21	19	40
missing	0	0	0
median	4.10	12.90	7.75
mean	8.95	20.35	14.36
SD	10.12	21.08	17.06

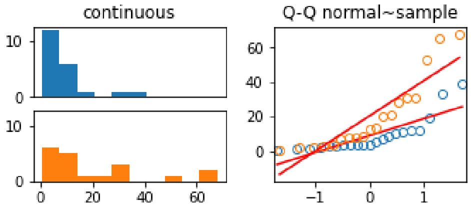

test	p-value
Levene	0.037
Welch's t	0.042
Mann-Whitn	0.058

Rutherford ~ Group

	ACT	ST	total
n	21	19	40
missing	0	0	0
median	5.00	5.00	5.00
mean	4.90	5.00	4.95
SD	0.30	0.33	0.32

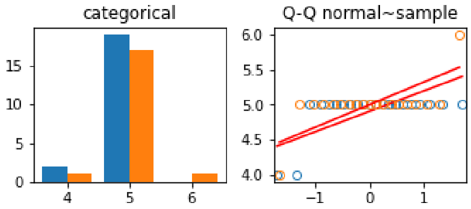

test	p-value
Levene	0.92
Student's t	0.35
Mann-Whitn	0.36

Graziani ~ Group

	ACT	ST	total
n	21	19	40
missing	0	0	0
median	5.00	5.00	5.00
mean	5.19	5.26	5.22
SD	0.81	0.56	0.70

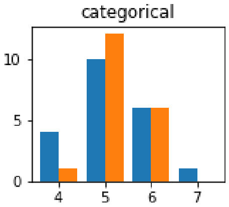

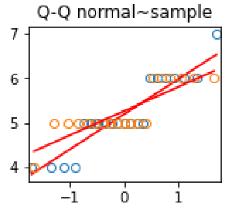

test	p-value
Levene	0.25
Student's t	0.75
Mann-Whitn	0.72

WIfI_clin ~ Group

	ACT	ST	total
3	6 (15%)	3 (8%)	9 (22%)
4	15 (38%)	16 (40%)	31 (78%)
total	21 (52%)	19 (48%)	40 (100%)
missing	0	0	0

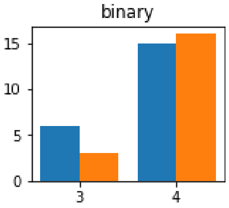

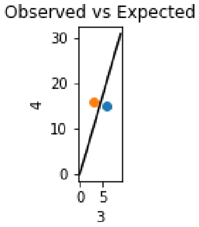

test	p-value
χ^2 Pearson	0.33
χ^2 Yates	0.56
Fisher exact	0.46
odds ratio	2.13

WIfI_wound ~ Group

	ACT	ST	total
n	21	19	40
missing	0	0	0
median	2.00	2.00	2.00
mean	1.90	1.95	1.93
SD	0.54	0.52	0.53

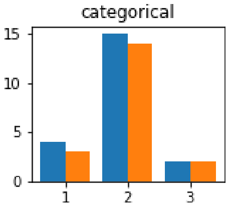

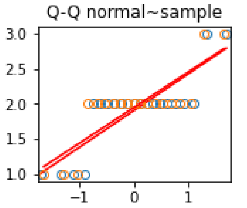

test	p-value
Levene	0.88
Student's t	0.80
Mann-Whitn	0.81

WIfI_ischemia ~ Group

	ACT	ST	total
3	21 (52%)	19 (48%)	40 (100%)
total	21 (52%)	19 (48%)	40 (100%)
missing	0	0	0

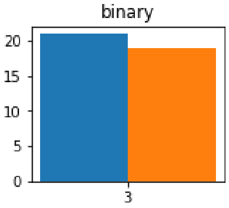

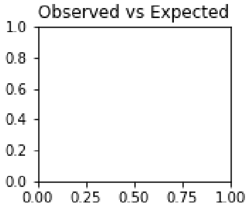

test	p-value

WIfI_infection ~ Group

	ACT	ST	total
n	21	19	40
missing	0	0	0
median	0.0	1.00	1.00
mean	0.52	0.79	0.65
SD	0.60	0.71	0.66

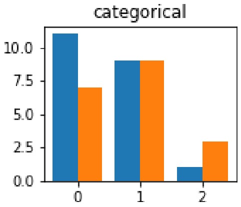

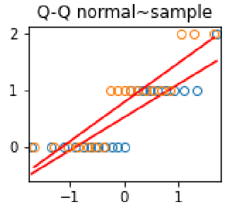

test	p-value
Levene	0.99
Student's t	0.21
Mann-Whitn	0.24

```
[8]:                   formula        scale          test         p
0              CRP ~ Group   continuous  Mann-Whitney  0.057964
1       Rutherford ~ Group  categorical  Mann-Whitney  0.362337
2         Graziani ~ Group  categorical  Mann-Whitney  0.717200
3        WIfI_clin ~ Group       binary  Fisher exact  0.457040
4       WIfI_wound ~ Group  categorical  Mann-Whitney  0.808658
5   WIfI_ischemia ~ Group       binary          None       NaN
6  WIfI_infection ~ Group  categorical  Mann-Whitney  0.236653
```

0.4 TcPO2 table

```
[9]: ll = []
for week in [0, 4, 12, 24]:
    st = df.query('Group == "ST"')['02_' + str(week)].dropna()
    act = df.query('Group == "ACT"')['02_' + str(week)].dropna()
    ll.append({
        'week' : week,
        'ST n' : st.shape[0],
        'ST mean' : f'{st.mean():.1f} ± {st.std(ddof=1):.1f}',
        'ACT n' : act.shape[0],
        'ACT mean' : f'{act.mean():.1f} ± {act.std(ddof=1):.1f}',
        'ACT_mean - ST_mean' : f'{act.mean() - st.mean():.1f}',
#           'SEM' : s.sem(),
#           'CI' : s.sem() * stats.t.ppf(1-alpha/2, n-1)
    })
table3 = pd.DataFrame(ll).set_index('week')
table3
```

```
[9]:       ST n      ST mean  ACT n     ACT mean ACT_mean - ST_mean
week
0        19  21.1 ± 11.4     21   18.7 ± 9.9               -2.4
4        17  22.7 ± 17.2     20  35.5 ± 17.5               12.8
12       13  20.1 ± 13.9     16  41.9 ± 18.3               21.8
24       13  41.9 ± 14.8     15  44.7 ± 14.2                2.8
```

0.5 TcPO2 figure

```
[10]: # prepare data for plotting
ll = []
for treatment in ['ST', 'ACT']:
    data = df.query('Group == @treatment')
    for week in [0, 4, 12, 24]:
        s = data['02_' + str(week)].dropna()
        n = s.shape[0]
        bs = stats.bootstrap([s], np.mean).confidence_interval
        ll.append({
            'treatment' : treatment,
```

```
            'week' : week,
            'n' : n,
            'mean' : s.mean(),
            'CI' : s.sem() * stats.t.ppf(1-alpha/2, n-1),
            'ci_low' : bs.low,
            'ci_high' : bs.high,
        })
O2 = pd.DataFrame(ll)
O2
```

[10]:

	treatment	week	n	mean	CI	ci_low	ci_high
0	ST	0	19	21.105263	5.488172	15.578947	25.631579
1	ST	4	17	22.705882	8.821060	15.000000	30.705882
2	ST	12	13	20.076923	8.396793	12.692308	27.230769
3	ST	24	13	41.923077	8.934092	31.066331	47.692308
4	ACT	0	21	18.666667	4.511557	14.380952	22.809524
5	ACT	4	20	35.550000	8.204456	28.400000	43.550000
6	ACT	12	16	41.875000	9.742059	33.000000	50.250000
7	ACT	24	15	44.733333	7.846929	38.200000	52.214607

[11]:

```
# plot
gg = (ggplot(O2)
 + aes(x='week', y='mean', color='treatment', fill='treatment', label='n',␣
↪ymin='ci_low', ymax='ci_high')
 + scale_color_cmap_d('tab10')
 + scale_fill_cmap_d('tab10')
 + geom_ribbon(alpha=.3, color=None)
 + geom_line(size=1)
 + geom_point()
 + geom_text(nudge_x=-0.8, nudge_y=1, size=9)
 + annotate('segment', x=0, xend=0, y=4, yend=8, color='C0',␣
↪arrow=arrow(length=.1))
 + annotate('text', x=0, y=2, color='C0', label='ACT')
 + annotate('segment', x=12, xend=12, y=4, yend=8, color='C1',␣
↪arrow=arrow(length=.1))
 + annotate('text', x=12, y=2, color='C1', label='ACT')
 + scale_x_continuous(breaks = range(0, 25, 4))
 + labs(color='Group', fill='Group', y=r'$TcPO_2 [mm Hg]$', x='Time [weeks]')
 + coord_cartesian(ylim=(0, None))
 + theme_light()
 + theme(panel_grid=element_blank())
)
gg
```

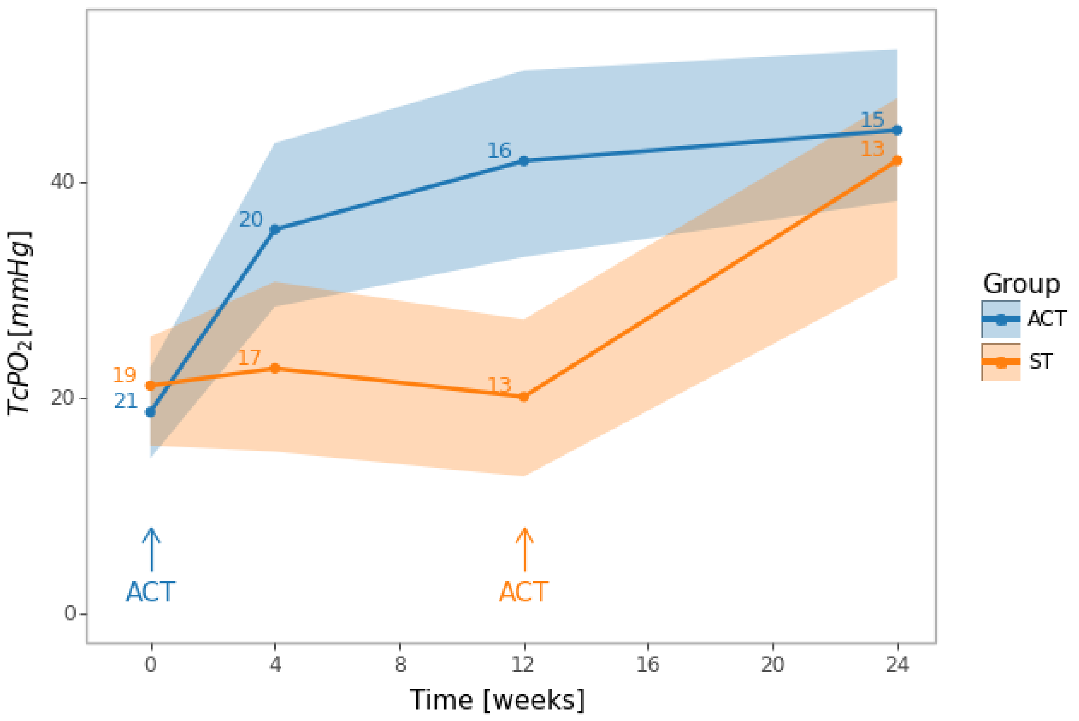

[11]: <ggplot: (8729631154673)>

[12]:
```
gg.save('gitignore/TcPO2.tiff', dpi=300)
```

```
/opt/conda/envs/py3.9/lib/python3.9/site-packages/plotnine/ggplot.py:719:
PlotnineWarning: Saving 6.4 x 4.8 in image.
/opt/conda/envs/py3.9/lib/python3.9/site-packages/plotnine/ggplot.py:722:
PlotnineWarning: Filename: gitignore/TcPO2.tiff
```

0.6 TcPO2 family hypothesis testing

- unpaired tests

[13]:
```
unpaired = si.compare(['O2_12', 'O2_24'], 'Group', df)
unpaired
```

O2_12 ~ Group

	ACT	ST	total
n	16	13	29
missing	5	6	11
median	42.00	17.00	33.00
mean	41.88	20.08	32.10
SD	18.28	13.90	19.58

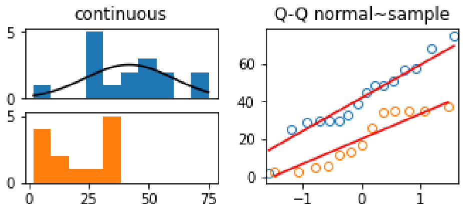

test	p-value
Levene	0.48
Student's t	0.0015
Mann-Whitn	0.0084

O2_24 ~ Group

	ACT	ST	total
n	15	13	28
missing	6	6	12
median	48.00	45.00	46.50
mean	44.73	41.92	43.43
SD	14.17	14.78	14.26

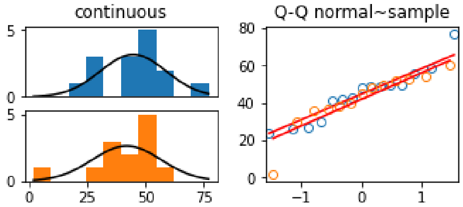

test	p-value
Levene	0.97
Student's t	0.61
Mann-Whitn	0.91

[13]:

```
          formula       scale          test         p
0  O2_12 ~ Group  continuous  Mann-Whitney  0.008428
1  O2_24 ~ Group  continuous             t  0.612283
```

- paired tests

[14]:

```
ll = []
for group in ['ST', 'ACT']:
    data = df.query('Group == @group')
    res = si.compare([['O2_0', 'O2_12'], ['O2_12', 'O2_24']], data=data,␣
↪parametric=False)
    res['formula'] = group + ': ' + res['formula']
    ll.append(res)
paired = pd.concat(ll)
paired
```

O2_0 vs. O2_12

	O2_0	O2_12
n	13	13
missing	6	6
median	23.00	17.00
mean	20.69	20.08
SD	11.14	13.90

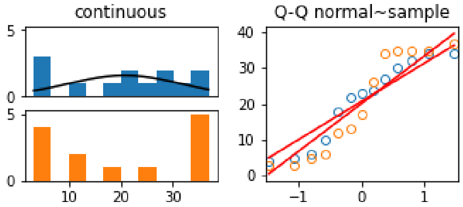

test	p-value
paired t	0.89
rank-sum	0.86

O2_12 vs. O2_24

	O2_12	O2_24
n	13	13
missing	6	6
median	17.00	45.00
mean	20.08	41.92
SD	13.90	14.78

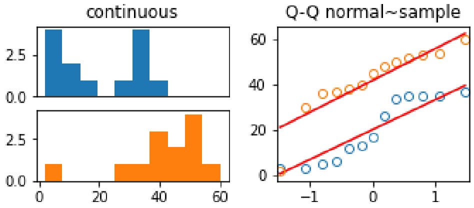

test	p-value
paired t	0.00054
rank-sum	0.00086

O2_0 vs. O2_12

	O2_0	O2_12
n	16	16
missing	5	5
median	19.50	42.00
mean	20.81	41.88
SD	9.61	18.28

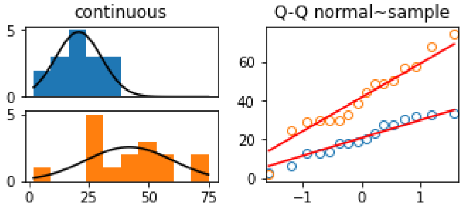

test	p-value
paired t	0.00056
rank-sum	0.00085

O2_12 vs. O2_24

	O2_12	O2_24
n	15	15
missing	6	6
median	45.00	48.00
mean	42.67	44.73
SD	18.64	14.17

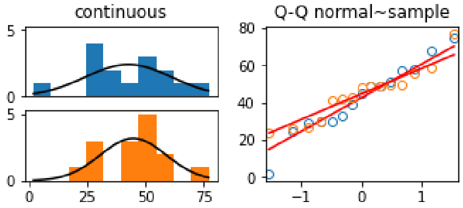

test	p-value
paired t	0.64
rank-sum	0.92

[14]:

```
                 formula       scale      test         p
0    ST: O2_0 vs. O2_12  continuous  rank-sum  0.857555
1   ST: O2_12 vs. O2_24  continuous  rank-sum  0.000858
0   ACT: O2_0 vs. O2_12  continuous  paired t  0.000564
1  ACT: O2_12 vs. O2_24  continuous  paired t  0.641846
```

[15]:

```
tests = pd.concat([unpaired, paired]).rename({'p':'uncorrected p'}, axis=1)
corr = sm.stats.multitest.multipletests(tests['uncorrected p'], method='holm',␣
 ↪alpha=0.05)
tests['corrected p'] = corr[1]
tests['significant'] = corr[0]
tests.reset_index(drop=True)
```

[15]:

```
                 formula       scale          test  uncorrected p  corrected p  \
0          O2_12 ~ Group  continuous  Mann-Whitney       0.008428     0.033712
1          O2_24 ~ Group  continuous             t       0.612283     1.000000
2    ST: O2_0 vs. O2_12  continuous      rank-sum       0.857555     1.000000
3   ST: O2_12 vs. O2_24  continuous      rank-sum       0.000858     0.004291
4   ACT: O2_0 vs. O2_12  continuous      paired t       0.000564     0.003381
5  ACT: O2_12 vs. O2_24  continuous      paired t       0.641846     1.000000

   significant
0         True
1        False
2        False
3         True
4         True
5        False
```

0.7 Secondary outcomes

[16]:
```
secondary = []
```

[17]:
```
secondary.append(si.compare_one('Healed_12', 'Group', df))
```

Healed_12 ~ Group

	ACT	ST	total
0.0	11 (38%)	13 (45%)	24 (83%)
1.00	5 (17%)	0 (0%)	5 (17%)
total	16 (55%)	13 (45%)	29 (100%)
missing	5	6	11

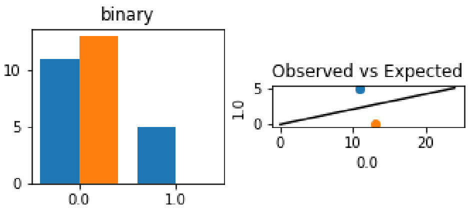

test	p-value
χ^2 Pearson	0.027
χ^2 Yates	0.085
Fisher exact	0.048
odds ratio	0.0

[18]:
```
secondary.append(si.compare_one('WAR', 'Group', df))
```

WAR ~ Group

	ACT	ST	total
n	17	13	30
missing	4	6	10
median	12.30	0.80	6.05
mean	12.14	0.75	7.21
SD	5.22	1.71	7.01

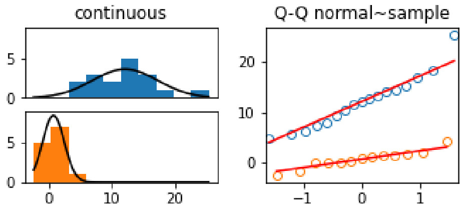

test	p-value
Levene	0.0088
Welch's t	4.7e-08
Mann-Whitn	3.7e-06

[19]:
```
secondary.append(si.compare_one('Minor_amp', 'Group', df))
```

Minor_amp ~ Group

	ACT	ST	total
0.0	15 (41%)	12 (32%)	27 (73%)
1.00	5 (14%)	5 (14%)	10 (27%)
total	20 (54%)	17 (46%)	37 (100%)
missing	1	2	3

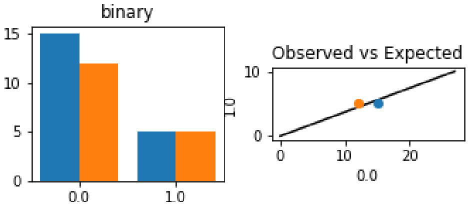

test	p-value
χ^2 Pearson	0.76
χ^2 Yates	1.00
Fisher exact	1.00
odds ratio	1.25

[20]:
```
secondary.append(si.compare_one('Amp_12', 'Group', df))
```

Amp_12 ~ Group

	ACT	ST	total
0.0	16 (43%)	13 (35%)	29 (78%)
1.00	4 (11%)	4 (11%)	8 (22%)
total	20 (54%)	17 (46%)	37 (100%)
missing	1	2	3

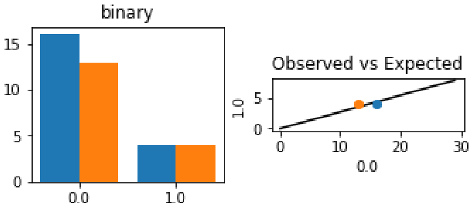

test	p-value
χ^2 Pearson	0.79
χ^2 Yates	1.00
Fisher exact	1.00
odds ratio	1.23

[21]:
```
secondary.append(si.compare_one('AFS_12', 'Group', df))
```

AFS_12 ~ Group

	ACT	ST	total
0.0	4 (11%)	4 (11%)	8 (22%)
1.00	16 (43%)	13 (35%)	29 (78%)
total	20 (54%)	17 (46%)	37 (100%)
missing	1	2	3

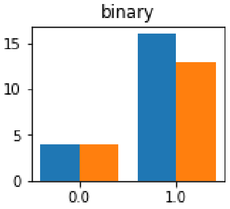

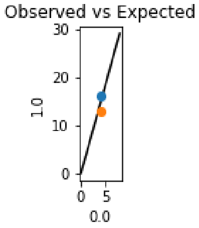

test	p-value
χ^2 Pearson	0.79
χ^2 Yates	1.00
Fisher exact	1.00
odds ratio	0.81

[22]:
```
si.compare_one('VAS_0', 'Group', df);
```

VAS_0 ~ Group

	ACT	ST	total
n	21	19	40
missing	0	0	0
median	5.00	6.00	5.50
mean	5.76	5.58	5.67
SD	2.43	2.17	2.28

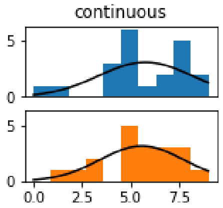

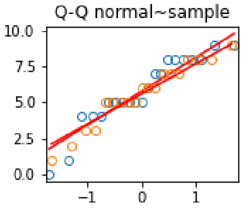

test	p-value
Levene	0.58
Student's t	0.80
Mann-Whitn	0.80

[23]:
```
secondary.append(si.compare_one('VAS_12', 'Group', df))
```

VAS_12 ~ Group

	ACT	ST	total
n	16	13	29
missing	5	6	11
median	2.00	6.00	4.00
mean	2.25	5.85	3.86
SD	2.11	1.72	2.64

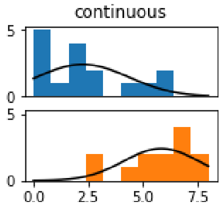

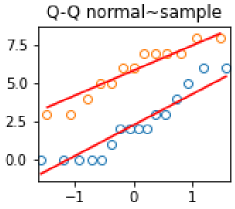

test	p-value
Levene	0.51
Student's t	3.6e-05
Mann-Whitn	0.00022

[24]:
```
si.compare_one('EQ-5D_0', 'Group', df);
```

EQ-5D_0 ~ Group

	ACT	ST	total
n	21	19	40
missing	0	0	0
median	45.00	45.00	45.00
mean	47.95	46.16	47.10
SD	12.00	8.85	10.53

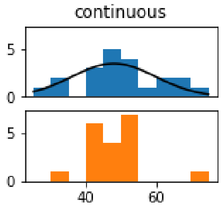

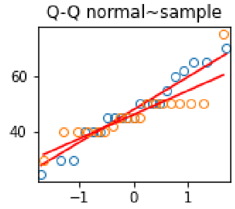

test	p-value
Levene	0.15
Student's t	0.60
Mann-Whitn	0.52

```
[25]: secondary.append(si.compare_one('EQ-5D_12', 'Group', df))
```

EQ-5D_12 ~ Group

	ACT	ST	total
n	16	13	29
missing	5	6	11
median	69.00	40.00	60.00
mean	69.56	41.77	57.10
SD	13.23	9.61	18.20

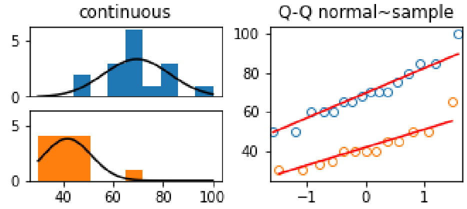

test	p-value
Levene	0.34
Student's t	8.9e-07
Mann-Whitn	2.4e-05

```
[26]: si.compare_one('SF-36-VB_0', 'Group', df);
```

SF-36-VB_0 ~ Group

	ACT	ST	total
0	2 (5%)	0 (0%)	2 (5%)
1	19 (48%)	19 (48%)	38 (95%)
total	21 (52%)	19 (48%)	40 (100%)
missing	0	0	0

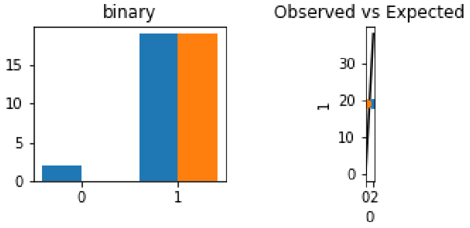

test	p-value
χ^2 Pearson	0.17
χ^2 Yates	0.51
Fisher exact	0.49
odds ratio	inf

```
[27]: secondary.append(si.compare_one('SF-36-VB_12', 'Group', df))
```

SF-36-VB_12 ~ Group

	ACT	ST	total
0.0	16 (55%)	0 (0%)	16 (55%)
1.00	0 (0%)	13 (45%)	13 (45%)
total	16 (55%)	13 (45%)	29 (100%)
missing	5	6	11

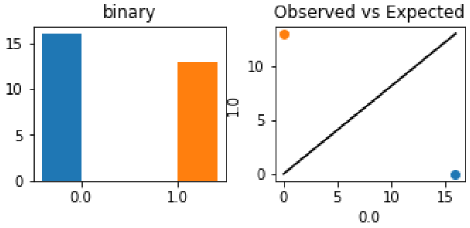

test	p-value
χ^2 Pearson	7.2e-08
χ^2 Yates	5.4e-07
Fisher exact	1.5e-08
odds ratio	inf

```
[28]: ll = []
      for var in [['VAS_0', 'VAS_12'], ['EQ-5D_0', 'EQ-5D_12']]:
          for group in ['ACT', 'ST']:
              data = df.query('Group == @group')
              ll.append(si.compare_one(var, data=data, parametric=False, group=group))
      paired_secondary = pd.DataFrame(ll)
```

VAS_0 vs. VAS_12

	VAS_0	VAS_12
n	16	16
missing	5	5
median	5.00	2.00
mean	5.50	2.25
SD	2.53	2.11

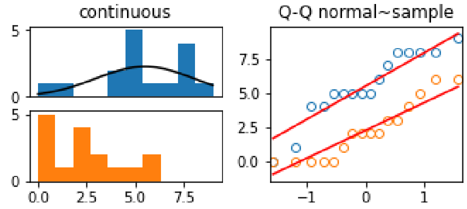

test	p-value
paired t	3.4e-05
rank-sum	0.0016

VAS_0 vs. VAS_12

	VAS_0	VAS_12
n	13	13
missing	6	6
median	5.00	6.00
mean	5.00	5.85
SD	2.24	1.72

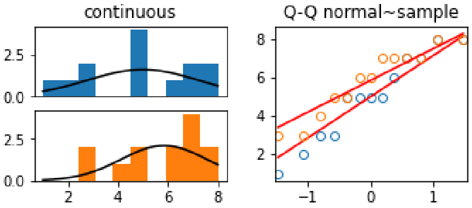

test	p-value
paired t	0.0025
rank-sum	0.34

EQ-5D_0 vs. EQ-5D_12

	EQ-5D_0	EQ-5D_12
n	16	16
missing	5	5
median	50.00	69.00
mean	50.75	69.56
SD	11.79	13.23

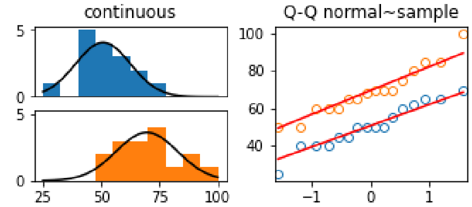

test	p-value
paired t	0.00052
rank-sum	0.00053

```
/home/mkah/statistikem/statistikem.py:259: UserWarning: ['EQ-5D_0', 'EQ-5D_12']:
all measurements possiblylognormal. Tests not implemented, yet!
```

EQ-5D_0 vs. EQ-5D_12

	EQ-5D_0	EQ-5D_12
n	13	13
missing	6	6
median	45.00	40.00
mean	47.08	41.77
SD	10.22	9.61

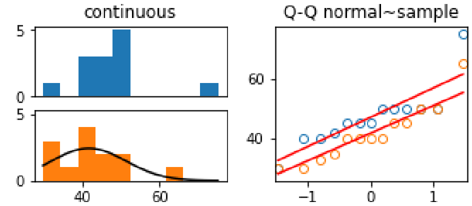

test	p-value
paired t	0.0060
rank-sum	0.11

[29]:
```
so = pd.concat([pd.DataFrame(secondary), paired_secondary])
so['p-value'] = so['p'].apply(lambda p: format(p, '.3f'))
so
```

[29]:

	formula	scale	test	p	p-value
0	Healed_12 ~ Group	binary	Fisher exact	4.761905e-02	0.048
1	WAR ~ Group	continuous	t	4.651029e-08	0.000
2	Minor_amp ~ Group	binary	Fisher exact	1.000000e+00	1.000
3	Amp_12 ~ Group	binary	Fisher exact	1.000000e+00	1.000
4	AFS_12 ~ Group	binary	Fisher exact	1.000000e+00	1.000
5	VAS_12 ~ Group	continuous	t	3.594171e-05	0.000
6	EQ-5D_12 ~ Group	continuous	t	8.927129e-07	0.000
7	SF-36-VB_12 ~ Group	binary	Fisher exact	1.473537e-08	0.000
0	VAS_0 vs. VAS_12	continuous	rank-sum	1.649411e-03	0.002

```
1      VAS_0 vs. VAS_12  continuous      paired t  2.481915e-03   0.002
2  EQ-5D_0 vs. EQ-5D_12  continuous      paired t  5.237496e-04   0.001
3  EQ-5D_0 vs. EQ-5D_12  continuous      rank-sum  1.062274e-01   0.106
```

0.8 Limb survival

[30]:
```
amp = df[['Patient', 'Group', 'Months_amp', 'Amp_12', 'Minor_amp', 'DO_FU',␣
↪'DO_reason']].copy()
amp['amp_or_death'] =  amp['Amp_12']
amp['duration'] = amp['Months_amp']
amp.loc[df['Amp_12'] == 0.0, 'duration'] = np.nan
amp['duration'].fillna(12.1, inplace=True)
amp = amp.query('Patient not in [4, 9, 40]').reset_index(drop=True)
amp
```

[30]:
```
    Patient Group  Months_amp  Amp_12  Minor_amp  DO_FU   DO_reason  \
0         2   ACT         NaN     0.0        0.0    NaN         NaN
1         3   ACT         NaN     0.0        0.0    NaN         NaN
2         6   ACT         NaN     0.0        1.0    NaN         NaN
3         8   ACT         NaN     0.0        0.0    NaN         NaN
4        10   ACT         NaN     0.0        1.0    NaN         NaN
5        13   ACT         NaN     0.0        0.0    NaN         NaN
6        14   ACT         3.0     1.0        0.0   12.0  amputation
7        17   ACT         7.0     0.0        1.0    NaN         NaN
8        16   ACT         NaN     0.0        0.0    NaN         NaN
9        18   ACT         2.0     1.0        0.0   12.0  amputation
10       20   ACT         3.0     1.0        0.0   12.0  amputation
11       21   ACT         NaN     0.0        0.0    NaN         NaN
12       23   ACT         1.0     1.0        0.0   12.0  amputation
13       25   ACT         NaN     0.0        1.0    NaN         NaN
14       29   ACT         NaN     0.0        0.0    NaN         NaN
15       30   ACT         NaN     0.0        0.0    NaN         NaN
16       33   ACT         5.0     0.0        1.0   24.0  amputation
17       34   ACT         NaN     0.0        0.0    NaN         NaN
18       35   ACT         NaN     0.0        0.0    NaN         NaN
19       36   ACT         NaN     0.0        0.0    NaN         NaN
20        1    ST         NaN     0.0        1.0    NaN         NaN
21        5    ST         NaN     0.0        0.0    NaN         NaN
22        7    ST         NaN     0.0        1.0    NaN         NaN
23       11    ST         NaN     0.0        0.0    NaN         NaN
24       12    ST         2.0     1.0        0.0   12.0  amputation
25       15    ST         6.0     0.0        1.0    NaN         NaN
26       19    ST         2.0     1.0        0.0   12.0  amputation
27       22    ST         1.0     1.0        0.0    4.0  amputation
28       24    ST         NaN     0.0        0.0    NaN         NaN
29       26    ST         NaN     0.0        1.0    NaN         NaN
30       27    ST         NaN     0.0        0.0    NaN         NaN
```

31	28	ST	1.0	1.0	0.0	12.0	amputation
32	31	ST	NaN	0.0	0.0	NaN	NaN
33	32	ST	9.0	0.0	0.0	NaN	NaN
34	37	ST	NaN	0.0	0.0	NaN	NaN
35	38	ST	NaN	0.0	1.0	NaN	NaN
36	39	ST	NaN	0.0	0.0	NaN	NaN

	amp_or_death	duration
0	0.0	12.1
1	0.0	12.1
2	0.0	12.1
3	0.0	12.1
4	0.0	12.1
5	0.0	12.1
6	1.0	3.0
7	0.0	12.1
8	0.0	12.1
9	1.0	2.0
10	1.0	3.0
11	0.0	12.1
12	1.0	1.0
13	0.0	12.1
14	0.0	12.1
15	0.0	12.1
16	0.0	12.1
17	0.0	12.1
18	0.0	12.1
19	0.0	12.1
20	0.0	12.1
21	0.0	12.1
22	0.0	12.1
23	0.0	12.1
24	1.0	2.0
25	0.0	12.1
26	1.0	2.0
27	1.0	1.0
28	0.0	12.1
29	0.0	12.1
30	0.0	12.1
31	1.0	1.0
32	0.0	12.1
33	0.0	12.1
34	0.0	12.1
35	0.0	12.1
36	0.0	12.1

```
[31]: fig, ax = plt.subplots()
previous = []
models = []
for group in ['ACT', 'ST']:
    data = amp.query('Group == @group')
    kmf = lifelines.KaplanMeierFitter()
    kmf.fit(data['duration'], data['amp_or_death'], label=group)
    kmf.plot(ci_show=True, ax=ax)
    models.append(kmf)
    for baseline, baseline_label in previous:
        p = lifelines.statistics.logrank_test(
            baseline['duration'], data['duration'], baseline['amp_or_death'],␣
↪data['amp_or_death']).p_value
    previous.append((data, group))

lifelines.plotting.add_at_risk_counts(*models, rows_to_show=['At risk'], ␣
↪ax=ax) #, 'Censored', 'Events'
ax.set_ylabel('Amputation free survival [%]')
print(p)
at = matplotlib.offsetbox.AnchoredText(f'p = {p:.2f}', loc='upper right',␣
↪frameon=False)
ax.add_artist(at)
ax.set_ylim([0, 1.0])
y = np.linspace(0, 1, 6)
ax.set_yticks(y)
ax.set_yticklabels((y * 100).astype(int))
ax.set_xlabel('Time [weeks]')
ax.legend(loc='lower left')
```

```
0.7368686485762548
```

```
[31]: <matplotlib.legend.Legend at 0x7f0868eefe80>
```

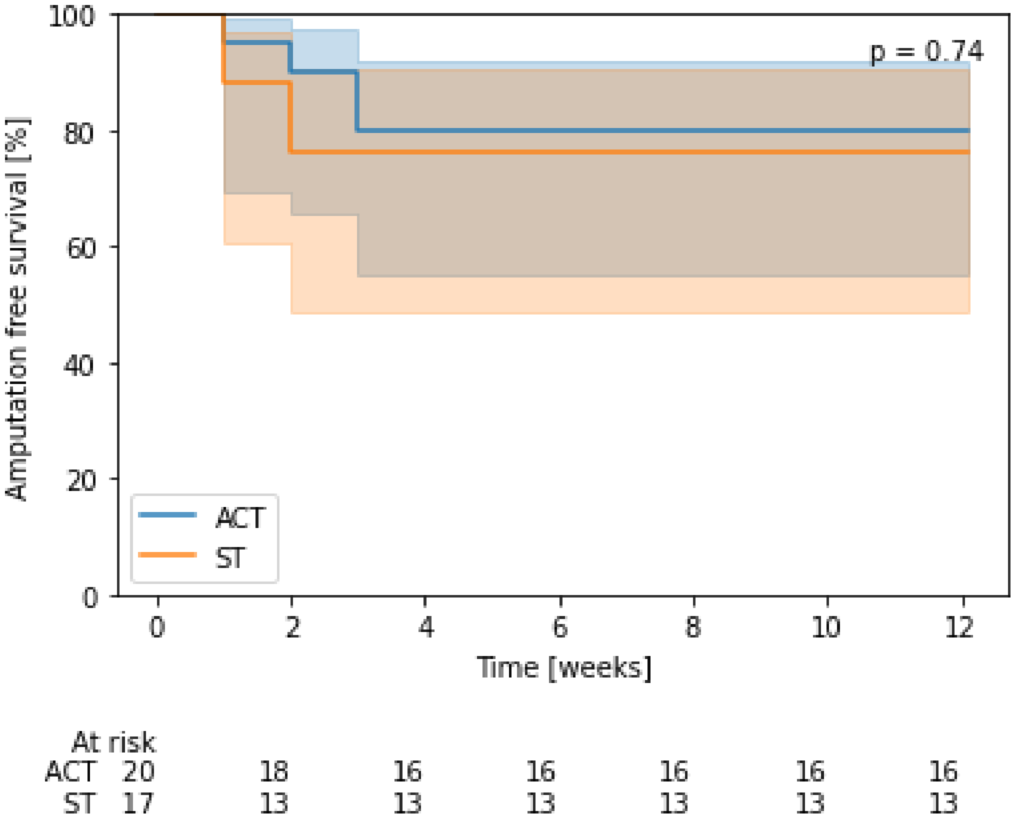

[38]:
```
plt.rc('savefig', facecolor='white')
fig.savefig('gitignore/survival.tiff', dpi=300, bbox_inches="tight")
```
